# Community engagement for vaccine delivery in low- and middle-income countries and humanitarian settings: A scoping umbrella review

**DOI:** 10.1371/journal.pgph.0006307

**Published:** 2026-04-24

**Authors:** Jonathan A. Polonsky, Rose Burns, Alex Odlum, Yashua Alkali Hamza, Yohannes Mulugeta, Sharon Abramowitz, Luisa Enria, Karl Blanchet

**Affiliations:** 1 Geneva Centre of Humanitarian Studies, University of Geneva, Geneva, Switzerland; 2 London School of Hygiene and Tropical Medicine, London, United Kingdom; 3 Childcare and Wellness Clinics, Abuja, Nigeria; 4 Haramaya University, Harar, Ethiopia; 5 Georgetown University, Washington, United States of America; Institute of Development Studies, UNITED KINGDOM OF GREAT BRITAIN AND NORTHERN IRELAND

## Abstract

Vaccination is a critical public health intervention in humanitarian and low- and middle-income country (LMIC) settings, where populations face heightened risks of vaccine-preventable diseases. Community Engagement (CE) offers a pathway to address challenges related to local ownership, trust, and acceptance, yet its effectiveness in these contexts remain underexplored. We conducted an umbrella scoping review of published reviews to synthesise evidence on the role of CE in vaccination delivery in humanitarian and LMIC contexts. Reviews were identified through searches of PubMed, Web of Science, Embase, and Google Scholar, screened in Covidence, and appraised using the Database of Abstracts of Reviews of Effects (DARE) criteria. Data were mapped against the International Federation of the Red Cross and Red Crescent Societies (IFRC) CE Impact Framework. Of 303 deduplicated studies, 39 met inclusion criteria. Most focused on routine childhood vaccines. CE activities were widespread, typically involving community participation, two-way communication, capacity strengthening, and feedback mechanisms. CE interventions were consistently associated with improved vaccine uptake, reduced hesitancy, and enhanced trust. Effective strategies included co-management of campaigns, engagement of religious and community leaders, culturally tailored communication, and school- or home-based delivery supported by trusted local figures. Multi-component interventions combining education, outreach, and digital tools were particularly effective. However, considerable variability existed in how CE was defined and operationalised across studies. CE should be recognised as a core pillar of vaccination strategy rather than an optional addition. It builds trust, fosters ownership, and addressed sociocultural barriers to access. Tailored, multi-component approaches leveraging trusted community figures are especially promising. Definitional ambiguity and inconsistent evaluation frameworks currently limit understanding of what works, for whom, and in what context. Future research should prioritise standardising CE definitions, developing context-sensitive theories of change, and strengthening methods for evaluating CE’s contribution to vaccine coverage and equity.

## Introduction

Vaccination against a range of priority pathogens is a crucial intervention for protecting the public health of populations affected by crises [1]. Indeed, ensuring adequate measles vaccination coverage is recognised as a priority intervention among newly-displaced children by several organisations, including Médecins Sans Frontières (MSF) and SPHERE, a network of humanitarian actors whose aim is to improve the quality of humanitarian assistance [2,3]. Vaccines can be a cost-effective approach to preventing and mitigating the impacts of infectious disease outbreaks, and reducing overall morbidity and mortality, the overarching aim of humanitarian health interventions.

However, humanitarian contexts present particular challenges to vaccine delivery, whether in the maintenance of routine vaccination or the roll-out of vaccination campaigns in response to outbreaks [1]. In particular, these include large-scale population movements, overburdened and/or dysfunctional health systems, damaged health and transport infrastructure, and security risks to health staff involved in vaccination. These are in addition to other challenges that are universal to vaccination in low- and middle-income countries (LMICs), including logistic considerations (such as the timely importation of sufficient quantities of vaccines and ensuring the cold chain from warehouse to the point of delivery in often remote, hot locations), and ensuring access to vaccination services for the most vulnerable and neglected persons (such as minority groups, young girls, and those residing in hard-to-reach areas). One dimension that is increasingly recognised as being critical to the success of vaccination efforts is the role of confidence, trust, and acceptance at both the community and individual levels. In recent years, numerous studies have documented the outsized role these play, and of the importance therefore of integrating *Risk Communication and Community Engagement* (RCCE) into efforts to ensure vaccine uptake.

*Community Engagement* (CE) is broadly understood as efforts to bring together “traditional, community, civil society, government, and opinion groups and leaders; and expanding collective or group roles in addressing the issues that affect their lives” [4]. It refers to the process of working collaboratively with and through groups of people affiliated by geographic proximity, special interest, or similar situations to address issues affecting the well-being of those people. In the context of vaccines, CE entails the active involvement of local communities - through consultation, participation, and partnership - in the planning, implementation, and evaluation of vaccination campaigns, through a broad range of activities. It emphasises building trust, promoting transparency, addressing cultural sensitivities, and enhancing community ownership of vaccination efforts, aiming to increase vaccine acceptance, address misinformation, and ensure equitable access to immunization services.

Despite this increasing recognition of the importance of CE in public health response, efforts to synthesise the evidence concerning the role of CE in vaccine delivery in humanitarian settings have been limited. Much of the existing work has either not addressed the specific application of CE to vaccination, overlooked the nuances of community dynamics, or has focused heavily on economic evaluations without capturing the social and participatory dimensions of CE. Furthermore, the concept of “community” has limitations in general, giving a semblance of unity and homogeneity that may be overly reductive and simplistic, particularly in certain humanitarian contexts, such as those that involve population displacement and a disruption to established community networks and structures [5 7].

Although global initiatives such as the READY Initiative’s *Monitoring and Evaluation (M&E) Planning Tool for Risk Communication and Community Engagement (RCCE)* [8] and the *Joint Evaluation of the Risk Communication and Community Engagement (RCCE) Collective Service* [9] have established structured indicators, outcome domains, and theories of change for assessing risk communication and community engagement in health emergencies, these frameworks have not yet been systematically applied to vaccination delivery in LMICs or humanitarian settings.

Recent literature has highlighted both the potential and the limitations of CE in vaccination efforts. Evidence has shown that CE can drastically improve vaccine acceptance and coverage rates. The use of behavioural and social approaches to enhance vaccine uptake has been found to effectively address vaccine hesitancy and foster trust in vaccination programmes [10,11]. Culturally appropriate strategies that incorporate CE have been shown to increase childhood immunization rates by aligning vaccination efforts with local cultural norms and involving community members directly [12]. High levels of community involvement have been associated with substantial improvements in vaccination rates [13]. These studies collectively suggest that CE is not just a supplementary component but should be thought of as a central strategy in achieving high vaccination coverage. However, none of these reviews focussed on humanitarian and/or LMIC settings. There is limited understanding of why such CE interventions might be effective, especially in the context of vaccination. Promising mechanisms of change for community engagement interventions in crisis response include the use of existing structures (e.g., known community and opinion leaders); community empowerment (training local people to be involved in the response); and enabling partnership and coordination which avoids duplication of efforts during the crisis [14].

Other reviews of community engagement interventions within the broad context of communicable disease control and health crisis responses did not specifically focus on vaccination efforts, leaving a gap in understanding CE’s direct impact on vaccine uptake [14–17]. Insights into the cost-effectiveness of health interventions have also been reported, but the effectiveness of CE interventions for vaccination was not addressed [18]. Finally, some studies focused on the costs associated with promoting vaccination, but these economic evaluations did not explore the broader social dynamics and trust-building that are integral to CE [18,19].

We addressed these evidence synthesis gaps by conducting an umbrella scoping review to describe the landscape of research that has been conducted in the past decade on CE for vaccination in humanitarian and LMIC settings.

## Materials and methods

We followed guidelines on umbrella and scoping reviews while conducting and reporting this research [20–22]. A protocol for this review was not prospectively registered prior to data extraction.

### Eligibility criteria

Reviews describing CE and vaccine delivery conducted in humanitarian crisis-affected settings and LMICs were eligible for inclusion.

We defined crisis-affected settings as those in which ‘an event or series of events has resulted in a critical threat to health, safety, security or well-being of a community or other large group of people’ [23], identifying five conditions, as previously described [24]: (1) progressive loss of livelihoods and deterioration of essential services due to ever-present risk of violence; (2) mass displacement into camp-like settlements; (3) displacement into neighbouring host communities; (4) sudden loss of livelihoods and rapid environmental change due to natural disaster, and (5) food crises. For practical purposes, this amounted to including those countries with humanitarian operation plans during the study period, as published in the OCHA Global Humanitarian Overviews for relevant years [25].

We also included LMICs because much of the evidence and lessons learned from these settings was assumed to apply also to the subset of these countries that have also experienced crises. Furthermore, the term “humanitarian settings” is somewhat intangible and hard-to-define.

We used the PICOS framework (population, intervention, comparison, outcome, study design) to develop inclusion and exclusion criteria, which are presented in detail in the Supporting information (S1 Table). Briefly, eligible articles were those in which the study population was people living in humanitarian settings or LMICs, the intervention was some form of CE, the outcome was a measure of vaccination coverage and/or community perception of vaccination activities, with comparison between groups receiving and those not receiving the intervention or between pre- and post-intervention phases. We restricted articles to reviews (both systematic and literature) written in English and published in peer reviewed journals between 2013 and 2024.

### Search strategy and information sources

A comprehensive literature search was initially conducted on 5 July 2024 in PubMed and Google Scholar for English-language publications dated between 2013 and 2024, using the complete search strategy detailed in the Supplementary Materials. The search was subsequently updated on 5 September 2025 to include studies published in any language, with no date restrictions up to July 2024, and was expanded to encompass the Web of Science and Embase databases.

Studies were imported into Covidence systematic review management software (Veritas Health Innovation) [26]. Entries were checked for duplication. The following steps were then carried out on the deduplicated records independently by two reviewers. Irrelevant articles were excluded in two-steps; first by screening titles and abstracts and then by screening the full text of the remaining articles. Discrepancies and borderline cases were resolved through discussions between at least two reviewers.

### Data charting process

The final list of included articles was divided among reviewers for data charting (extraction and summary) using a structured questionnaire. The data items extracted related to two domains: (1) study metadata: authors, publication year, the years during which the interventions were implemented, the countries of study, and study quality; (2) study detail: vaccine(s) studies, intervention description, intervention impact, and key findings. For charting the intervention description and impact, we mapped the CE activities and outcomes against those listed in an evaluation framework developed by the International Federation of the Red Cross and Red Crescent Societies (IFRC) (S1 Fig, Supporting information).

### Critical appraisal of individual sources of evidence

The quality of the evidence of the included studies was assessed using the Database of Abstracts of Reviews of Effects (DARE) criteria [27] adapted by Cairn’s et al [28], with each rated “High”, “Medium”, or “Low”. The DARE criteria assess whether a review includes a clear research question, an adequate search strategy, appropriate inclusion criteria, a quality assessment of included studies, and a valid synthesis of findings. Studies rated “High” met all or nearly all criteria; those rated “Medium” met most criteria with minor concerns; and those rated “Low” had notable methodological gaps. These qualitative scores were taken into consideration at the data synthesis stage, with greater caution applied when drawing conclusions supported predominantly by low-rated reviews.

### Synthesis of results

We performed a qualitative synthesis of the findings, according to the vaccines of interest, the interventions employed, and the relationship of these interventions with different measures of outcome. Findings were grouped according to the interventions and outcomes described in the IFRC evaluation framework.

We synthesised and summarised identifiable trends and commonalities in the findings and highlighted important gaps and limitations in the published research. We report our findings according to the Preferred Reporting Items for Systematic Reviews and Meta-Analyses extension for scoping reviews (PRISMA-ScR) statement [29]. The completed checklist is presented in Supporting information (S1 Checklist). Given the substantial variability in how CE was defined and operationalised across the included reviews, we also developed a working definition and conceptual typology of CE inductively from the synthesised evidence, presented at the end of the Results section.

## Results

### Selection of sources of evidence

**Fig 1:**
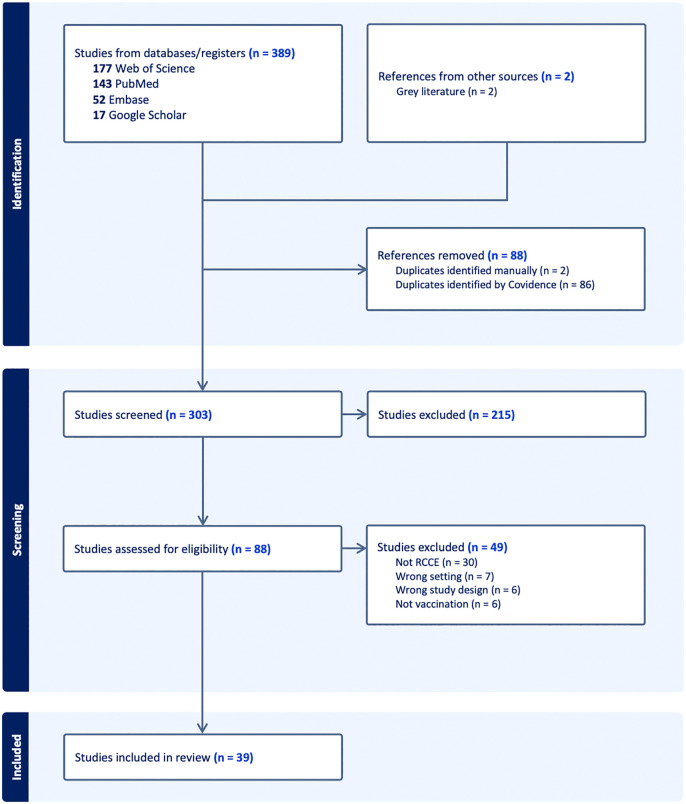
PRISMA 2020 flow diagram depicting the study screening and inclusion process.

The database search identified 391 studies. After removing 88 duplicates, nearly three-quarters of the remaining 303 articles (215, 71.0%) were excluded at the abstract screening stage for not meeting the inclusion criteria, leaving 88 for full text screening. Of these, 49 (55.7%) were excluded: 30 did not feature a CE intervention, seven were not in LMICs or humanitarian settings, six were not reviews, and six were not concerned with vaccination. This left 39 reviews for inclusion [30–68] (Fig 1).

### Characteristics of sources of evidence

#### Characteristics of publications and setting.

**Fig 2:**
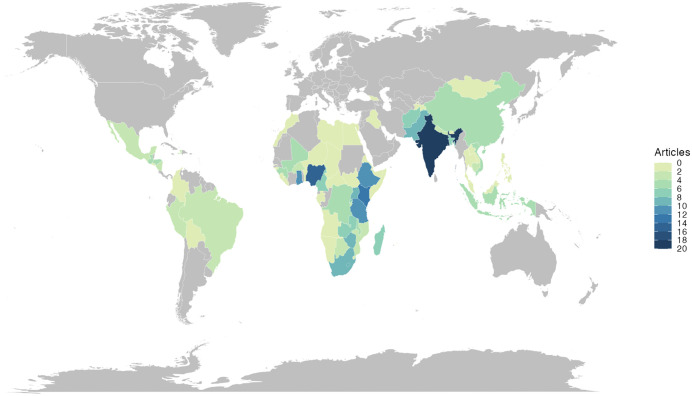
Map of the number of review articles meeting the inclusion criteria, by country. N.B. **Reviews may include research relating to multiple countries.** Map base layer data are provided by Natural Earth (https://www.naturalearthdata.com/http//www.naturalearthdata.com/download/110m/cultural/ne_110m_admin_0_countries.zip).

Within the included reviews, studies were conducted in 76 countries across Sub-Saharan Africa, Asia, South and Central America, and Middle East and North Africa (Fig 2). The countries in which the most research was conducted were India (19 articles), Nigeria (16 articles), Kenya (14 reviews), and Ethiopia, Ghana, and Tanzania (11 reviews each). Two reviews that were based on the same search strategy covered multiple LMICs but did not list the countries [46,61].

#### Characteristics of research topics.

**Fig 3:**
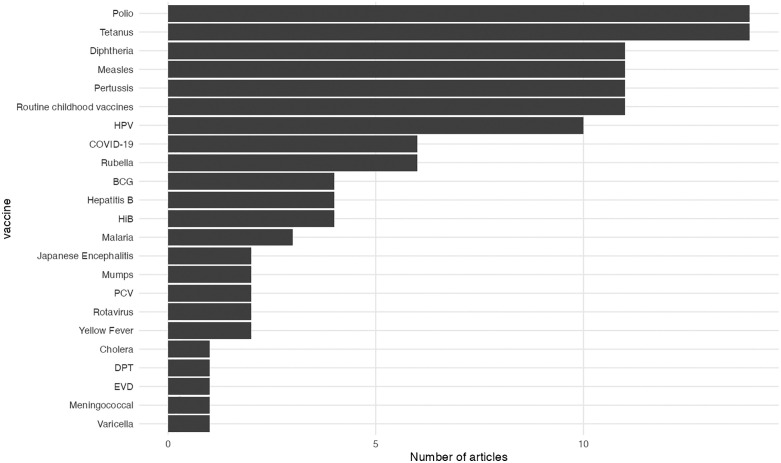
Number of review articles meeting the inclusion criteria, by vaccine of interest. N.B. Reviews may include research relating to multiple antigens.

Vaccines: Research focussed on routine childhood vaccines, with specific mention of polio and tetanus in 14 articles, diphtheria, measles, and pertussis in 11, and Human papillomavirus (HPV) in ten (Fig 3). A further 15 vaccines were featured (COVID-19, rubella, Calmette-Guérin (BCG), hepatitis B, Haemophilus influenzae type b (Hib), malaria, Japanese encephalitis, mumps, pneumococcal conjugate vaccine (PCV), rotavirus, yellow fever, cholera, EVD, meningococcal, and varicella), with the number of articles featuring each vaccine ranging from six to one.

Thirty reviews were about routine vaccination exclusively, while four were concerned solely with outbreak response vaccination, specifically COVID-19 [54,63,68] and EVD [44]. A further five reviews addressed both routine and outbreak response vaccination [30,31,48,57,66].

**Fig 4:**
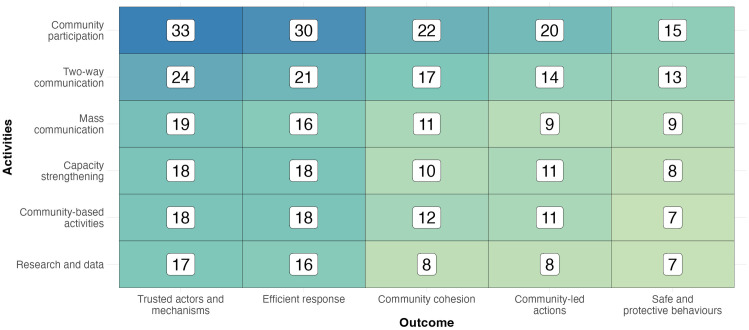
Heatmap of the number of review articles meeting the inclusion criteria, by intervention and outcome measure. **N.B.** Review articles may contain research that relates to multiple interventions, impacts, and/or combinations thereof.

Activities: Most reviews (34, 87%) included “Community participation” as an activity (Fig 4). “Two-way communication” (25, 64%) was included in one-third of the reviews, while “Capacity strengthening”, “Community-based activities”, and “Mass communication” (each 19, 49%) were each included in half of the reviews. “Research and data” was included in 17 (44%) reviews. The remaining item from the analytical framework, “Coordination and advocacy”, did not feature in any reviews.

Outcomes: “Trusted actors and mechanisms” (23, 96%) was the most frequently used measure of outcome, appearing in all-but-one (38, 97%) of the articles, with “Efficient response” featuring in 34 (87%) articles (Fig 4). “Community cohesion” (24, 62%) and “Community-led actions” (21, 54%) were included in most articles, while “Safe and protective behaviours” was included in 18 (46%) articles.

#### Critical appraisal within sources of evidence.

The search returned a mix of literature, scoping, and systematic reviews, of varying quality. There were 19 literature reviews, 16 systematic reviews, and four scoping reviews. Sixteen articles were rated “High” quality, 18 “Medium”, and five “Low”, with the rationale behind each score presented in Supporting information (S2 Table).

**Fig 5:**
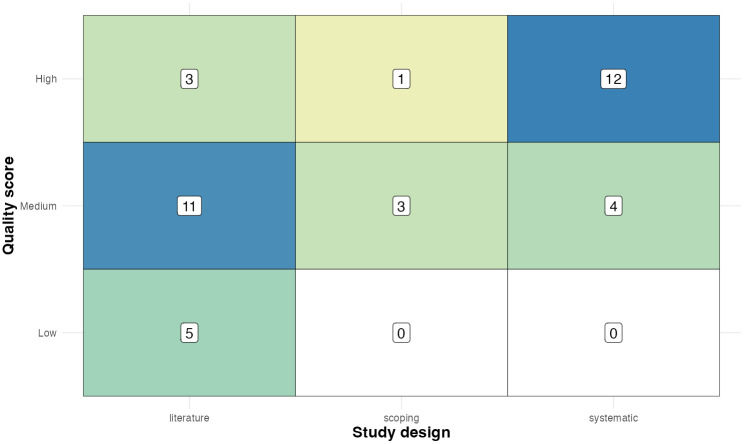
Heatmap of the number of review articles meeting the inclusion criteria, by study design and quality rating.

Three-quarters (12, 75%) of the systematic reviews were given quality scores of “High”, with the remaining four rated as “Medium” (Fig 5). Three of the four scoping reviews were rated as “Medium”, with one rated as “High”. As may be anticipated, the literature reviews were generally rated as lower quality compared to the systematic reviews, with just three (16%) rated as “High”, 11 (58%) as “Medium”, and the remaining five (26%) as “Low”.

### Synthesis of results

**Fig 6:**
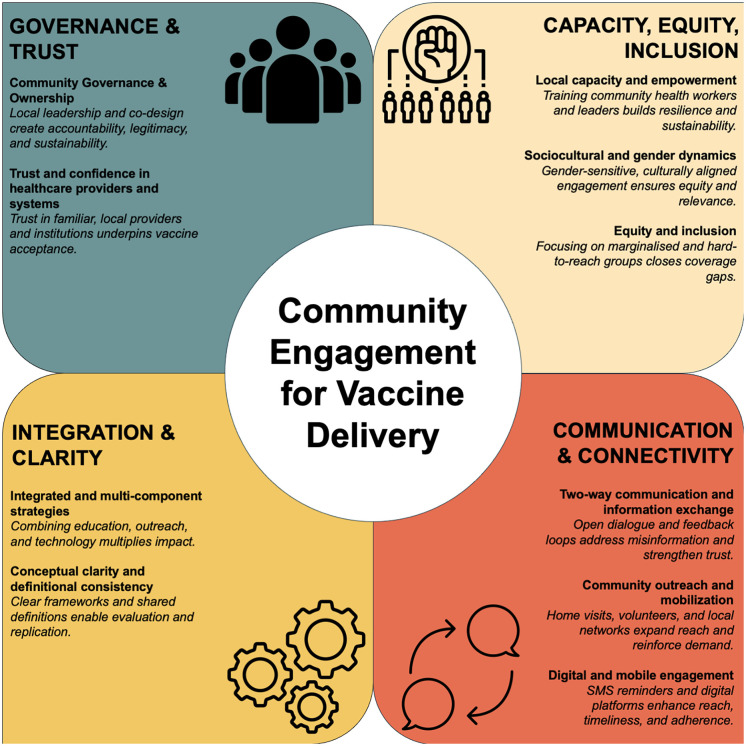
Conceptual framework illustrating ten thematic areas of community engagement (CE) for vaccine delivery in low- and middle-income and humanitarian settings. The themes are grouped into four interlinked domains which together represent a dynamic system through which CE fosters trust, equity, and improved vaccine uptake.

Overall, the studies generally suggested that CE interventions play an important role in enhancing vaccination uptake and fostering positive attitudes towards vaccination. These findings clustered around ten thematic areas within four overarching domains that together describe the core mechanisms through which CE operates and the conditions that enable it to influence vaccination delivery, acceptance, and sustainability (Fig 6). The full extraction table is presented in Supporting information (S3 Table).

#### Domain 1: Governance & Trust.

1. Community governance and ownership

Community involvement, engagement with leadership, and local ownership were reported to be essential components of successful vaccination programmes. These factors ensured that initiatives were co-created, embraced, and sustained by communities themselves rather than imposed by external actors. Co-management and co-design approaches, where communities had meaningful input in planning and execution, enhanced ownership and long-term sustainability [43,49,50,65]. Establishing community health committees and engaging respected community or religious leaders empowered local populations to advocate for and maintain vaccination programmes [31,38,42,44,48,58,61,68].

Education and involvement of traditional and Islamic religious leaders helped overcome resistance and improve uptake in low-literacy settings. Such leaders wield influence within their communities and can serve as powerful advocates [31,62,68]., Conversely, opposition from influential institutions, such as the Catholic Church in Kenya, undermined confidence in HPV vaccination [39,42,51,63]. These findings highlight the central role of leadership in shaping norms and attitudes around vaccination. Sustained engagement with teachers, parents, and local governance structures was also crucial in maintaining community buy-in, momentum, and accountability [56,59,64].

1. Trust and confidence in healthcare providers and systems

Trust in proximate, familiar actors rather than in distant institutions was a decisive factor in vaccine acceptance. Confidence in local healthcare providers, teachers, and community health workers emerged as one of the strongest predictors of adherence to vaccination recommendations [41,43,47,51,64]. For example, women in Haiti were more likely to accept HPV vaccination when recommended by trusted healthcare professionals, and in school-based programmes, teachers were key influencers encouraging parental consent [47].

Building and maintaining trust between healthcare systems and communities was particularly important where misinformation or vaccine hesitancy were prevalent. When healthcare providers demonstrated transparency, respect, and reliability, they became central to countering fears and restoring confidence in vaccines.

#### Domain 2: Capacity, equity, inclusion.

1. Local capacity and empowerment

Strengthening local capacity, particularly through the training of healthcare workers and community leaders, was identified as having a positive effect on improving vaccination coverage, by helping communities take ownership of vaccination efforts, improving both their quality and reach.

Empowering healthcare professionals with the skills necessary to communicate effectively and address vaccine hesitancy was particularly important in low-resource settings [32,36,48]. Building local capacity ensured the resilience of vaccination programmes, particularly in conflict settings, where strong local leadership and system-building were essential for maintaining vaccination efforts [55].

In addition to training healthcare workers, strengthening local leadership and community health systems contributed to long-term sustainability. Empowering local systems helped foster accountability and ensured that vaccination efforts were better integrated into broader public health frameworks [46]. This also ensured that communities could take ownership of vaccination programmes, improving both reach and quality [34,43,68].

1. Sociocultural and gender dynamics

Local norms and social hierarchies strongly shaped vaccine acceptance and required careful adaptation of engagement strategies. Gender roles and decision-making patterns, in particular, influenced whether caregivers could access vaccination services [33,34,42]. In many contexts, such as Ethiopia and Kenya, women’s limited autonomy restricted their ability to seek health services independently, often resulting in missed vaccination opportunities [38,45]. Engaging fathers and other male household decision-makers helped mitigate these constraints and improve coverage [38,45].

Tailoring interventions to local cultural expectations and belief systems increased the perceived relevance of vaccination programmes and reduced resistance [30,39–43,50,52,63]. Religious and traditional leaders again played a vital role, lending credibility to vaccination efforts and framing them within locally accepted moral and social narratives.

Social media and mass communication were double-edged: when content was culturally attuned and accessible to different literacy levels, it expanded reach and awareness [30,41,51,63]; when poorly managed, it amplified misinformation and scepticism [51].

Overall, we found that the question of power was not effectively addressed in the articles. As others have shown, the use of the term ‘community’ suggests homogeneity and thus obscures diversity and the workings of power between different social groupings across localities [6]. Where power dynamics were described in the included reviews, they tended to focus on the positive role of community and religious leaders as facilitators of CE, rather than on the ways in which existing power structures can constrain or distort it. Efforts to map power and trust, including in informal influencers, has been shown in other contexts to yield more effective, stratified engagement strategies [5].

Gender hierarchies were the most consistently documented power dynamic. Women’s limited autonomy and low decision-making power at the household level were identified as barriers to vaccination across multiple settings, with women in some contexts unable to vaccinate children without spousal approval and facing significant domestic consequences for doing so [38,45,64]. Fathers’ refusal to vaccinate was also explicitly named as a barrier in Nigeria [52], and gendered norms prevented female health workers from engaging male household members in some settings [64]. In Haiti, social norms and patriarchal healthcare structures meant women’s decisions were strongly shaped by the approval of male partners and family networks [47].

Beyond gender, religious and cultural leaders were found to act as gatekeepers to community access, and a lack of political commitment at national or regional level undermined CE efforts on the ground [53]. Evidence from the Ebola response illustrated how failing to work through existing power structures initially generated distrust, while channelling information through respected community leaders was more effective [44]. These findings suggest that CE interventions are shaped by existing power structures within communities, and that without accounting for these, engagement risks reaching only the most visible or vocal groups rather than the most marginalised.

1. Equity and inclusion

Equity and inclusion are key features for reaching underserved and marginalized populations during vaccination programmes. Equity-focused interventions helped ensure that vulnerable groups, particularly in rural and hard-to-reach areas, were included in vaccination campaigns [31,46,63]. Community feedback mechanisms were important in tailoring programmes to local needs and ensuring that vaccination efforts were responsive to the unique challenges faced by different population groups, particularly addressing logistical barriers to access [41,52]. Involving vulnerable groups from the outset ensured the effectiveness and sustainability of vaccination campaigns [61].

In conflict and post-conflict settings, addressing structural inequalities was key to rebuilding trust in healthcare systems and improving vaccine outcomes [53]. Strengthening local health systems and addressing barriers to access, such as geographic isolation and socioeconomic disadvantage, were successful strategies for ensuring equity in vaccination efforts.

An important equity dimension insufficiently addressed across the included reviews is the situation of zero-dose children (ZDC), those who have not received a single vaccine dose and who are disproportionately concentrated among the most marginalised populations. Coverage of zero-dose and unvaccinated children was identified as a key evidence gap, having been assessed in only one systematic review, with calls for greater attention to this group in future research [46]. While ZDC were not the specific focus of some reviews, it was noted that barriers to reaching them may differ from those facing partially vaccinated children, and that CE alone may be insufficient without addressing structural determinants such as health service availability, with explicit calls for subgroup analyses focused on ZDC [49,50]. ZDC were the explicit focus of at least one included review, which referenced GAVI’s zero-dose strategy and noted the inability to distinguish barriers for ZDC from those for dropouts as a key limitation [62]. The scale of the problem in humanitarian settings was underscored by empirical data reporting zero-dose rates of 46.6% among Ethiopian IDPs and 29.9% among refugees [67], and approximately 3.1 million Nigerian children estimated to be zero-dose or missed-dose [66]. The CE strategies documented across the included reviews, including community mapping, home visits, engagement of marginalised groups, and social mobilisation, are in fact those most relevant to reaching ZDC, even where the term was not used explicitly. Future reviews and primary research should explicitly frame CE strategies for zero-dose communities as a priority equity lens, particularly in fragile and conflict-affected settings.

#### Domain 3: Communication & connectivity.

1. Two-way communication and information exchange

Effective communication was central to addressing knowledge gaps, mis- and dis-information, and concerns surrounding vaccination [41,56,65]. Establishing two-way communication channels between health authorities and communities facilitated the real-time identification and resolution of issues [38,44,48].

Communication strategies were most successful when short, focused, and audience-specific, improving retention and completion rates compared with longer, generalized sessions [30,42,51,58]. For example, brief, facility-based education for mothers in rural Pakistan increased adherence to follow-up vaccination visits [42].

Tailored, context-appropriate communication delivered through multiple channels, including community meetings, printed materials, and radio, helped clarify misconceptions and strengthen vaccine confidence [34,41,42,50,56,58,60,63,65]. Coordinated advocacy involving trusted figures further improved credibility, particularly in countering misinformation propagated via social media during the COVID-19 pandemic [42,43,47,51,54].

1. Community outreach and mobilisation

Direct outreach and social mobilisation were consistently associated with improved vaccination coverage, especially in underserved or hard-to-reach populations [31,33,40,42,45,67].

Home visits, door-to-door engagement, and personal communication with caregivers dispelled myths, reinforced key messages, and facilitated access to vaccination services [30,34,42,64,66]. In Uttar Pradesh, India, intensive social mobilisation correlated with greater booth attendance, higher perceived polio risk, and a substantial reduction in refusals, with nearly 80% of initially resistant families accepting OPV after follow-up visits [31].

Community volunteers and local women’s groups (such as Ethiopia’s Health Development Army), proved instrumental in fostering demand and bridging service gaps [42,43,45]. Mobilizing communities to participate in vaccination efforts was also a key factor in improving COVID-19 vaccine coverage [54]. School- and home-based vaccination models further improved access for adolescents and caregivers who might otherwise have been missed, particularly when teachers were engaged as trusted partners [39,49,51]. However, such programmes also required efforts to reach children not enrolled in schools, where a mix of social mobilisation and community engagement using community health workers played a critical role in bridging gaps, especially among marginalized communities [39,41,51].

1. Digital and mobile engagement

Digital and mobile tools expanded the channels through which communities could be reached and engaged. SMS reminders and other mHealth technologies improved timeliness and completion of vaccination schedules by providing caregivers with convenient, real-time prompts [37,42,54,59]. In Zimbabwe, localized text messages reduced delays and increased coverage, although in Guatemala, where baseline coverage was already high, SMS reminders had less impact [42].

Despite contextual variation, digital approaches offer a low-cost, scalable complement to traditional engagement methods, particularly in remote or resource-constrained settings [37]. As mobile phone access continues to expand globally, digital interventions are expected to play an increasingly important role in public health, including for vaccination campaigns targeting hard-to-reach populations [54].

#### Domain 4: Integration & clarity.

1. Integrated and multi-component strategies

Integrated interventions that combined education, outreach, and digital tools produced the strongest and most sustained improvements in vaccination uptake [30,42,57,59,63]. These multi-component strategies addressed multiple barriers simultaneously, such as misinformation, logistical challenges, and social resistance. For instance, packages that linked health education with community mobilisation and digital reminders improved coverage and reduced dropout rates [42], while those incorporating cash transfers further incentivized vaccination [57,59]. To ensure sustainability, future implementation should identify which combinations yield the greatest impact relative to cost [42].

1. Conceptual clarity and definitional consistency

While CE was consistently emphasised as a critical factor in the success of vaccination programmes, its definition and scope often varied considerably, leading to confusion and challenges during implementation, as has been previously reported [69]. Several reviews pointed out that CE encompassed a wide array of activities, ranging from basic communication efforts to more participatory roles, such as co-management and decision-making with local communities [35,42]. However, without clear frameworks or guidelines, the interpretation of CE became inconsistent, making it difficult to determine what actions truly fell under this umbrella term.

The importance of context-specific definitions was also noted, as CE could mean different levels of involvement depending on the setting. In some contexts, activities were limited to one-way information-sharing, while in others, it involved deeper, collaborative efforts that empowered local stakeholders to influence decision-making processes. This ambiguity contributed to confusion over what activities should be prioritized and how to best involve communities [49–51]. The variation in the roles of actors, such as teachers or CHWs, further highlighted the need for frameworks that clearly define activities, roles, and the extent of participation to ensure effective CE across diverse contexts.

To address this ambiguity, we have developed a working definition and conceptual typology of CE for vaccine delivery in LMICs and humanitarian settings, derived from recurrent patterns observed across the included studies. Our proposed working definition is: *CE in vaccine delivery refers to planned, sustained processes that meaningfully involve community members and their representatives in shaping demand, access, delivery, oversight, and evaluation of immunization services, with consequential influence on how vaccination is organised and experienced in practice.* Our conceptual typology identifies seven categories of engagement that form a spectrum from most to least direct community involvement (Box 1).

**Box 1. Typology of community engagement in vaccine delivery (N.B. many interventions were multi-pronged).**

**Type of Engagement**

Co-production of programming
Community participation & establishment of community advocacy groups
Two-way dialogue
Outreach and mobilisation
Communication and health promotion
Debunking misinformation
Mass communication/ digital engagement

## Discussion

### Summary of evidence

We found that community involvement and engagement of leaders are foundational pillars for the success of vaccination programmes. These elements help ensure that immunization efforts are not just imposed from external sources but are embraced and championed by the communities themselves. Sustained communication targeting key members of the community ensures continued community buy-in and ownership of vaccination efforts. By fostering a sense of ownership among these stakeholders, vaccination programmes can gain traction and appear to achieve higher levels of acceptance and coverage.

Early community involvement and social mobilisation are important for laying the groundwork for successful vaccination initiatives. Prior to the rollout of vaccination campaigns, comprehensive sensitization campaigns can help inform the population about the importance and safety of vaccines. These campaigns often leverage community leaders, teachers, and health workers as trusted sources of information to disseminate key messages effectively.

Effective communication and education strategies help address various challenges and concerns surrounding vaccination. One crucial aspect is establishing two-way communication channels that allow for the exchange of information and feedback between health authorities and the community. This appears to facilitate the identification and addressing of knowledge gaps, misinformation, and concerns in real-time.

Communication strategies should be tailored to the specific needs and concerns of the target population. This may involve utilizing various channels such as face-to-face communication, informational materials, and media campaigns. Moreover, efforts should focus on dispelling myths and addressing misconceptions about vaccines while emphasising their benefits and importance for public health.

Localised trust in people directly known to communities, rather than generic trust in governments, systems, news sources, or institutions, was shown to be important. This critical finding diverges from much current research on trust and is an important factor explaining part of *why* CE works when it does.

Vaccination programmes must be designed with the socio-cultural context and power dynamics of the target communities in mind. Interventions should be adapted to fit the local context and cultural norms of the communities they serve. This requires a critical appraisal of existing practices and experiences to ensure that vaccination initiatives are perceived as relevant and beneficial rather than imposing alien practices.

No single CE approach is sufficient on its own. The evidence points consistently to the value of combining strategies: mobile health tools such as SMS reminders extend reach and improve timeliness; school- and home-based delivery improves access for those who might otherwise be missed; and community volunteers bridge the gap between health services and resistant or hard-to-reach populations.

When these elements are combined, the effects appear to be greater than the sum of their parts, with multi- component approaches appearing to create an enabling environment that sustains behaviour change over time.

Education delivery methods should be informed by the preferences and needs of the target audience. Short, focused messages have been shown to be more effective in retaining information and influencing behaviour compared to longer, more generalized sessions.

Vaccine acceptance is shaped by a complex mix of individual, social, and structural factors. Confidence in vaccine safety, desire to protect one’s family, and trust in healthcare providers were among the most cited facilitators. Barriers included concerns about side effects, misinformation, and limited access to services, with structural constraints such as geographic isolation and weak supply chains compounding individual-level hesitancy. CE is particularly well-placed to address the social and individual dimensions of these barriers, though structural factors require parallel investment in health systems and infrastructure.

While these insights highlight the importance of community involvement and tailored communication strategies, the unique challenges faced in humanitarian contexts must be factored in. In such settings, time is often limited, there is immense pressure to act urgently, and sustained financing remains uncertain. These constraints make it challenging to implement prolonged and comprehensive CE strategies. In such settings, these lessons should be adapted to balance the urgency of response with community needs. For example, this could entail identifying key community representatives early on and focusing on rapid, targeted communication that can still foster a degree of ownership without requiring extensive resources or time.

Future research should focus on systematically evaluating the effectiveness and cost-effectiveness of CE interventions using mixed-methods frameworks that capture not only coverage and timeliness but also community trust, vaccine confidence, and the mechanisms through which CE exerts its effects. A key challenge is measuring the contextual impact of CE, which primarily creates an enabling environment for vaccine uptake rather than delivering vaccines directly. To address this, evaluation approaches should adopt a social-ecological perspective (rather than a traditional monitoring and evaluation approach), complemented by a clearly articulated theory of change for CE in vaccine delivery. Such a framework would map the pathways linking inputs, processes, and outcomes, clarify causal mechanisms and contextual influences, and identify where adaptation or additional support may be needed to strengthen the effectiveness and resilience of CE interventions.

Existing global frameworks provide a useful foundation for this work. The READY Initiative’s Monitoring and Evaluation (M&E) Planning Tool for Risk Communication and Community Engagement (RCCE) (8) offers a structured set of input, output, outcome, and impact indicators spanning domains such as trust, participation, rumour management, and feedback use. Similarly, the *Joint Evaluation of the Risk Communication and Community Engagement (RCCE) Collective Service (9)* defines outcome areas and a theory of change linking strengthened coordination, data use, and local capacity to improved CE quality and public health outcomes. Adapting and applying these frameworks to vaccination delivery in humanitarian and LMIC contexts could help move CE research from descriptive case studies toward systematic, comparable assessment of effectiveness.

In synthesising these findings, it is important to remember that CE is just one facet of an effective vaccination strategy. Consideration should also be given to such diverse topics as identification of the appropriate target population, ensuring equitable access to vaccine supply and robust supply chain logistics, training and capacity-building of the healthcare workforce, and establishing surveillance to monitor implementation [11,55]. However, CE, a topic that has historically not been given adequate consideration, is an essential approach that must be integrated in all vaccination strategies, particularly those targeting neglected and under-served populations living in crisis settings.

### Limitations

The main limitations of this review relate to its design as a scoping umbrella review. A protocol was not prospectively registered prior to data extraction; while prospective registration is recommended to enhance transparency and reduce the risk of reporting bias, this review was conceived and initiated before a protocol was formally documented, and we acknowledge this as a limitation. The search strategy also evolved iteratively across two phases, with the initial search limited to PubMed and Google Scholar before expanding to Web of Science and Embase; while all records were subject to identical eligibility criteria and screening procedures, this staged approach introduces a potential for selection bias. We also restricted our search to peer-reviewed publications to ensure methodological consistency, quality appraisal, and replicability across the included reviews, and grey literature was not systematically searched. Given that CE in vaccine delivery is often documented in such sources, this omission may have resulted in the exclusion of relevant operational evidence. However, as many of the included reviews had already incorporated evidence from grey and programmatic sources, this limitation was partially mitigated. Future work should explicitly include grey literature searches and direct engagement with operational actors to ensure that implementation-level insights are systematically captured.

The quality of evidence found was mixed, and generally of a lower quality. This was often connected with study design, such that the literature reviews were generally rated to be of lower quality, which is to be expected. The inclusion of narrative literature reviews alongside systematic and scoping reviews was a deliberate methodological choice, consistent with the umbrella scoping review approach, which aims to capture the breadth of available evidence rather than restrict it to the highest-quality study designs. This is therefore not so much a limitation of our methodology as a reflection of the quality of the available published literature that formed the basis of our research. We also acknowledge that DARE, as a broad appraisal instrument, was not specifically designed for umbrella reviews and may not fully capture the range of quality of the diverse review types included, and we therefore urge caution in interpreting these ratings as a precise measure of evidence quality. This does, however, highlight the need for more and better-quality research on both the effects of CE in vaccination programmes and on what constitutes best practice.

Finally, an important limitation of this research is that, while all the reviews described examples of what CE could do, none discussed what CE *could not do*. There was limited investigation of how and why specific interventions might be effective. Further research is needed addressing these gaps, including the use of process evaluations, which would help address the finding of substantial conceptual ambiguity regarding what activities do and do not constitute CE.

## Conclusions

Community engagement is a central determinant of effective vaccine delivery, particularly in fragile or underserved settings. It helps overcome barriers such as distrust, logistical constraints, and resistance by fostering trust, ownership, and local leadership, ensuring that vaccination efforts are perceived as community-driven rather than externally imposed. Tailored communication through trusted figures, combined with participatory education and dialogue, can address fears and misinformation while building durable confidence in vaccines and health systems.

Successful CE is culturally attuned and context-specific: effective interventions respect local norms and governance structures and rely on open, two-way communication. Integrating low-cost digital tools such as SMS reminders and leveraging existing networks can further extend the reach and responsiveness of these strategies.

CE should be understood not as an adjunct but as a core pillar of vaccination strategy. Embedding communities as active partners, rather than passive recipients, enhances both the immediate effectiveness and long-term sustainability of vaccination programmes. Continued refinement of CE’s conceptual boundaries, evaluation metrics, and theory of change will be critical to realizing its full potential in advancing equitable and resilient vaccine coverage.

## Supporting information

S1 Text Full bibliographic search terms. (DOCX)

S1 Checklist Preferred Reporting Items for Systematic reviews and Meta-Analyses extension for Scoping Reviews (PRISMA-ScR). Licensed under CC BY 4.0 (https://creativecommons.org/licenses/by/4.0/).
(DOCX)

S1 Table PICOS inclusion and exclusion criteria. (DOCX)

S2 Table Quality assessment of included reviews. (DOCX)

S3 Table Data charting extraction table of key information from reviews meeting the inclusion criteria. (DOCX)

S1 Fig Community Engagement Impact Framework (*Source: International Federation of Red Cross and Red Crescent Societies (IFRC)).* (DOCX)
