# Supplementary material for: Community engagement for vaccine delivery in low- and middle-income countries and humanitarian settings: A scoping umbrella review
Source: PLOS Glob Public Health. 2026 Apr 24;6(4):e0006307. doi: 10.1371/journal.pgph.0006307 (PMC13108762; doi:10.1371/journal.pgph.0006307)
Supplement: S1 Text — (DOCX) [file pgph.0006307.s001.docx]

## S1 Text. Full bibliographic search terms

(“community engagement” OR “community participation” OR “stakeholder engagement” OR “social mobilisation” OR “social mobilization” OR “community capacity” OR “behaviour* change” OR participation OR communication OR “community mobilisation” OR “community mobilization” OR sensitization OR sensitisation OR “health promotion” OR “public engagement” OR “community led” OR “community driven” OR grassroots OR cocreation OR “risk communication”)

AND

vaccin*

AND

(“Developing Countries”

OR

Afghanistan OR “Guinea-Bissau” OR Somalia OR “Burkina Faso” OR “Korea” OR “South Sudan” OR “Burundi” OR “Liberia” OR “Sudan” OR “Central African Republic” OR “Madagascar” OR “Syrian Arab Republic” OR “Chad” OR “Malawi” OR “Togo” OR “Congo “ OR “Mali” OR “Uganda” OR “Eritrea” OR “Mozambique” OR “Yemen” OR “Ethiopia” OR “Niger” OR “Zambia” OR “Gambia” OR “Rwanda” OR “Guinea” OR “Sierra Leone”

OR

“Angola” OR “India” OR “Philippines” OR “Algeria” OR “Indonesia” OR “Samoa” OR “Bangladesh” OR “Iran “ OR “São Tomé and Principe” OR “Benin” OR “Kenya” OR “Senegal” OR “Bhutan” OR “Kiribati” OR “Solomon Islands” OR “Bolivia” OR “Kyrgyz Republic” OR “Sri Lanka” OR “Cabo Verde” OR “Lao PDR” OR “Tanzania” OR “Cambodia” OR “Lebanon” OR “Tajikistan” OR “Cameroon” OR “Lesotho” OR “Timor-Leste” OR “Comoros” OR “Mauritania” OR “Tunisia” OR “Congo” OR “Micronesia” OR “Ukraine” OR “Côte d’Ivoire” OR “Mongolia” OR “Uzbekistan” OR “Djibouti” OR “Morocco” OR “Vanuatu” OR “Egypt” OR “Myanmar” OR “Vietnam” OR “El Salvador” OR “Nepal” OR “West Bank and Gaza” OR “Eswatini” OR “Nicaragua” OR “Zimbabwe” OR “Ghana” OR “Nigeria” OR “Haiti” OR “Pakistan” OR “Honduras” OR “Papua New Guinea”)
