# Supplementary material for: Community engagement for vaccine delivery in low- and middle-income countries and humanitarian settings: A scoping umbrella review
Source: PLOS Glob Public Health. 2026 Apr 24;6(4):e0006307. doi: 10.1371/journal.pgph.0006307 (PMC13108762; doi:10.1371/journal.pgph.0006307)
Supplement: S1 Table — (DOCX) [file pgph.0006307.s003.docx]

## S1 Table: PICOS inclusion and exclusion criteria

|  | **Inclusion criteria** | **Exclusion criteria** |
| --- | --- | --- |
| **Population(s)** | Populations living in humanitarian settings or LIC | Populations not living in these settings |
| **Intervention(s)** | Community engagement strategies and programmes for vaccination |  |
| **Comparison(s)** | - No intervention  - Pre-intervention period |  |
| **Outcome(s)** | - Vaccination coverage  - Community perception of the intervention |  |
| **Study designs** | - Reviews  - Systematic reviews |  |
| **Date or language criteria** | - 1 January 2014 – 30 June 2024  - English language |  |
