# Supplementary material for: Community engagement for vaccine delivery in low- and middle-income countries and humanitarian settings: A scoping umbrella review
Source: PLOS Glob Public Health. 2026 Apr 24;6(4):e0006307. doi: 10.1371/journal.pgph.0006307 (PMC13108762; doi:10.1371/journal.pgph.0006307)
Supplement: S2 Table — (DOCX) [file pgph.0006307.s004.docx]

## S3 Table: Quality assessment of included reviews

| Authors | Well-Defined Question | Search Strategy | Inclusion/  Exclusion Criteria | Primary  Study  Designs | Quality Assessment | Synthesis | Multiple Authors | Score | Quality | Reasoning |
| --- | --- | --- | --- | --- | --- | --- | --- | --- | --- | --- |
| Agrawal et al. (2020) | Yes | Yes | No | No | No | Yes | Yes | 4 | Medium | Lacks clear criteria, no formal quality assessment, but strong question and synthesis. |
| Alum et al. (2025) | Yes | Yes | No | No | No | Yes | Yes | 4 | Medium | Narrative review with stated databases/keywords, but no formal eligibility rules or appraisal; narrative synthesis only. |
| Ansar et al. (2024) | Yes | Yes | Yes | Yes | No | Yes | Yes | 6 | High | Clearly framed acceptance question with database search and explicit inclusion; primary designs described; no formal quality appraisal; structured narrative synthesis. |
| Aslam et al. (2023) | Yes | Yes | No | No | No | Yes | Yes | 4 | Medium | Strong question and synthesis, but lacks clear criteria and no formal quality assessment. |
| Bello et al. (2011) | Yes | Yes | No | No | No | Yes | Yes | 4 | Medium | Review notes a PubMed search but provides no detailed criteria or quality appraisal; narrative synthesis. |
| Bilous et al. (1997) | Yes | No | No | No | No | Yes | Yes | 3 | Low | Experience report describing campaigns without systematic search, criteria, or appraisal; narrative synthesis across countries by multiple authors. |
| Black & Richmond (2018) | Yes | Yes | Yes | Yes | No | Yes | Yes | 5 | Medium | Comprehensive question and synthesis, but lacks quality assessment and study design clarity. |
| Dudeja et al. (2024) | Yes | Yes | Yes | Yes | Yes | Yes | Yes | 7 | High | Well-defined question, comprehensive search and criteria, MMAT quality appraisal, clear identification of study designs, and structured synthesis by multiple authors. |
| Engelbert et al. (2022) | Yes | Yes | Yes | Yes | No | Yes | Yes | 5 | Medium | Good search strategy and criteria, but lacks formal quality assessment and relies on narrative synthesis. |
| Ewongwo et al. (2024) | Yes | Yes | Yes | Yes | No | Yes | Yes | 5 | Medium | Labeled systematic review with PRISMA-style process and explicit inclusion/exclusion; no formal study-quality appraisal reported. |
| Eze et al. (2025) | Yes | Yes | Yes | Yes | Yes | Yes | Yes | 7 | High | Systematic review following PRISMA/PICO, predefined eligibility, CASP appraisal, and structured synthesis. |
| George et al. (2025) | Yes | Yes | Yes | Yes | Yes | Yes | Yes | 7 | High | PRISMA-guided search across five databases, SPIDER-based eligibility, MMAT quality assessment, and clear synthesis. |
| Guignard et al. (2019) | Yes | No | No | No | No | Yes | Yes | 3 | Low | Narrative expert review with a clear focus but no reported systematic search, inclusion criteria, or formal appraisal; thematic synthesis by multiple authors. |
| Guillaume et al. (2022) | Yes | Yes | Yes | Yes | No | Yes | Yes | 5 | Medium | Strong question and synthesis, but lacks formal quality assessment. |
| Ismail et al. (2022) | Yes | Yes | Yes | Yes | Yes | Yes | Yes | 6 | High | Comprehensive search strategy, quality assessment, and synthesis, with clear criteria. |
| Jain et al. (2022a) | Yes | Yes | Yes | Yes | Yes | Yes | Yes | 6 | High | Strong criteria, quality assessment, meta-analysis, and multiple authors involved. |
| Jain et al. (2022b) | Yes | Yes | Yes | Yes | Yes | Yes | Yes | 7 | High | Comprehensive search strategy, well-defined criteria, quality assessment performed, strong synthesis and meta-analysis. |
| Jain et al. (2024) | Yes | Yes | Yes | Yes | No | Yes | Yes | 5 | Medium | Good search strategy and criteria, but lacks formal quality assessment. |
| Kane et al. (2012) | Yes | No | No | No | No | Yes | Yes | 3 | Low | Narrative policy/implementation review without explicit search, eligibility, or appraisal methods; integrative narrative synthesis. |
| Karanja-Chege (2022) | Yes | Yes | No | No | No | Yes | No | 3 | Low | Lacks clear criteria, formal quality assessment, and multiple authors. |
| Lambo & Nagulesapillai (2012) | Yes | No | No | No | No | Yes | Yes | 3 | Low | Country-focused narrative review summarizing multiple data sources; no systematic methods or quality assessment described. |
| Majekodunmi et al. (2022) | Yes | Yes | Yes | Yes | Yes | Yes | Yes | 7 | High | Comprehensive search, criteria, quality assessment, and synthesis. |
| Naidoo et al. (2023) | Yes | Yes | Yes | Yes | Yes | Yes | Yes | 6 | High | Strong criteria, quality assessment, meta-analysis, and clear synthesis. |
| O’Rourke et al. (2023) | Yes | Yes | Yes | Yes | No | Yes | Yes | 5 | Medium | Well-defined question and strong search strategy, but lacked formal quality assessment. Narrative synthesis used. |
| Obregon et al. (2009) | Yes | Yes | No | No | No | Yes | Yes | 4 | Medium | Describes sources and approach to reviewing communication evidence, but no formal inclusion/exclusion or appraisal; narrative synthesis. |
| Oketch et al. (2023) | Yes | Yes | Yes | Yes | Yes | Yes | Yes | 6 | High | Comprehensive synthesis, formal quality assessment, and clear criteria. |
| Oliver-Williams et al. (2017) | Yes | Yes | Yes | Yes | Yes | Yes | Yes | 6 | High | Strong criteria, formal quality assessment, and multiple authors involved. |
| Omoniyi & Williams (2020) | Yes | Yes | Yes | Yes | Yes | Yes | Yes | 6 | High | Clear criteria, formal quality assessment, comprehensive synthesis, multiple authors. |
| Owoyemi et al. (2021) | Yes | Yes | Yes | Yes | No | Yes | Yes | 5 | Medium | Strong search strategy and criteria, lacks formal quality assessment. |
| Oyo-Ita et al. (2023) | Yes | Yes | Yes | Yes | Yes | Yes | Yes | 7 | High | Strong question, search strategy, inclusion/exclusion criteria, and quality assessment. Meta-analysis was used appropriately. |
| Parsekar et al. (2024) | Yes | Yes | Yes | Yes | No | Yes | Yes | 5 | Medium | Strong criteria and synthesis, but no formal quality assessment performed. |
| Reda et al. (2025) | Yes | Yes | Yes | No | No | Yes | Yes | 5 | Medium | Scoping review with defined databases, eligibility and framework-guided synthesis; no formal quality appraisal, as typical for scoping reviews. |
| Saeterdal et al. (2014) | Yes | Yes | Yes | Yes | Yes | Yes | Yes | 7 | High | Comprehensive quality assessment, search strategy, and synthesis. |
| Singh et al. (2018) | Yes | Yes | Yes | Yes | No | Yes | Yes | 5 | Medium | Strong search strategy, but lacks formal quality assessment and relies on narrative synthesis. |
| Sinuraya et al. (2024) | Yes | Yes | No | No | No | Yes | Yes | 4 | Medium | Clearly stated question with described search at narrative level; lacks explicit inclusion criteria and formal appraisal; narrative synthesis by multiple authors. |
| Tilahun et al. (2020) | Yes | Yes | Yes | Yes | No | Yes | Yes | 5 | Medium | Good search strategy and criteria, but no formal quality assessment performed. |
| Tsu et al. (2014) | Yes | Yes | Yes | Yes | No | Yes | Yes | 5 | Medium | Strong search strategy and synthesis, lacks formal quality assessment. |
| Vadrevu et al. (2024) | Yes | Yes | Yes | Yes | No | Yes | Yes | 6 | High | Methods specify corpus of experimental/quasi-experimental impact evaluations and analytic framework; no explicit critical appraisal reported, but clear designs and systematic mapping. |
| Vouking et al. (2017) | Yes | Yes | Yes | Yes | Yes | Yes | Yes | 7 | High | Strong search strategy, formal quality assessment, comprehensive inclusion/exclusion criteria. |
