# Supplementary material for: Community engagement for vaccine delivery in low- and middle-income countries and humanitarian settings: A scoping umbrella review
Source: PLOS Glob Public Health. 2026 Apr 24;6(4):e0006307. doi: 10.1371/journal.pgph.0006307 (PMC13108762; doi:10.1371/journal.pgph.0006307)
Supplement: S3 Table — (DOCX) [file pgph.0006307.s005.docx]

## S3 Table: Data charting extraction table of key information from reviews meeting the inclusion criteria.

| Reference | Countries | Dates | Vaccine(s) | Quality | Intervention | Outcome | Key findings |
| --- | --- | --- | --- | --- | --- | --- | --- |
| Agrawal et al. (2020) | India | 2015-2019 | Polio; Measles; Rubella | Medium | Community-based activities; Community participation | Efficient response; Trusted actors and mechanisms; Community cohesion; Safe and protective behaviours | Influence of social relationships & access to information through social media had impact on MR vaccination. Vaccine acceptance higher when offered at school & high among parents who trusted teachers and other school children as sources of information. However, acceptance lower among parents who trusted information from social media. Major hindering factors were inadequate knowledge about vaccination campaign, rumours about vaccine safety, sudden planning & under-preparedness at health system level. Major facilitating factor was healthcare professionals spreading awareness & increasing trust in vaccines. Suspicions towards newly introduced vaccines, & doubts about need to vaccinate against uncommon diseases. Important interventions: Involvement of local stakeholders; encouraging use of different mass media techniques to increase awareness & address myths about vaccines; employing reminder & follow-up services; organising training sessions for healthcare workers to enhance their communication & engagement skills.  Religious leaders: Controversies & false information negatively impacted vaccine confidence over last 20 years. Religious leaders & community influencers eventually actively fostered social mobilisation that led to successful elimination of polio. Religious leaders should be included when communicating about immunisation as have major impact on support/rejection.  HCW: Remain most trusted advisors concerning vaccination. HCW preparedness for discussing vaccination is key, esp. communication training.   School teachers: Recognised as trustworthy source of information by parents accepting vaccination. Schools & schoolteachers should be engaged in vaccination campaigns. |
| Ansar et al. (2024) | Ghana, Nigeria | 2012-2023 | Malaria | High | Two-way communication; Research and data | Trusted actors and mechanisms; Safe and protective behaviours | Acceptance generally high; tailored communication and engagement recommended to address concerns and regional disparities |
| Aslam et al. (2023) | Pakistan, India, Ghana, Nicaragua, Zimbabwe, Mali, Honduras | 2022 | Diphtheria; Tetanus; Pertussis; Polio; Routine childhood vaccines | Medium | Community participation; Capacity strengthening; Two-way communication; Research and data | Efficient response; Trusted actors and mechanisms; Community cohesion; Community-led actions | Community participation helps build trust and vaccine uptake. Two-way communication improves community engagement. Addressing misinformation is crucial for increasing vaccine acceptance. Strengthening local capacity prepares communities for future interventions. |
| Bilous et al. (1997) | Philippines, China, Laos, Mongolia, Vietnam, Cambodia | 1992-1995 | Polio | Low | Community participation; Community-based activities; Mass communication; Capacity strengthening | Efficient response; Community-led actions; Trusted actors and mechanisms | Success linked to high-level political backing, logistics detail, and locally appropriate mobilization |
| Black & Richmond (2018) | Angola, Botswana, Ethiopia, Kenya, Malawi, Mauritius, Mozambique, Namibia, Rwanda, Seychelles, South Africa, South Sudan, Swaziland, Tanzania, Uganda, Zambia, Zimbabwe | 2011 | HPV | Medium | Community-based activities; Community participation; Mass communication | Efficient response; Trusted actors and mechanisms; Community cohesion; Community-led actions | High coverage achieved with inclusion of strategy to reach out-of-school girls, using social mobilization & community engagement. Higher vaccine uptake associated with community follow-up of vaccinated girls & school-based programmes. Early community involvement & social mobilization cornerstone of Rwanda’s HPV vaccination program. Nationwide sensitization campaign prior to roll-out, with early distribution of information & education through community leaders, teachers, & health workers. School-based delivery more effective than facility-based (but school attendance very high in Rwanda). |
| Dudeja et al. (2024) | India | 2011-2021 | BCG; Polio; DPT; Measles; Rubella; Hepatitis B; HiB; Japanese Encephalitis; Tetanus; COVID-19 | High | Research and data; Community-based activities; Mass communication; Capacity strengthening | Efficient response; Safe and protective behaviours; Trusted actors and mechanisms | Engagement plus system tools effective; limited evidence for some newer logistics technologies; gaps outside children/pregnancy |
| Engelbert et al. (2022) | Multiple LMICs | 2021 | Diphtheria; Tetanus; Pertussis; Measles; Polio | Medium | Community-based activities; Capacity strengthening; Two-way communication; Community participation | Safe and protective behaviours; Efficient response; Community cohesion; Community-led actions | Equity and inclusion are key to reaching underserved populations. Two-way communication; Community participation mechanisms help tailor programs to local needs. Strengthening local systems ensures long-term success. Communication helps create trust and better outcomes. |
| Guignard et al. (2019) | Benin, Bolivia, Botswana, Burkina Faso, Cambodia, Cameroon, Chad, Ghana, Guatemala, India, Madagascar, Malawi, Mali, Mongolia, Niger, Peru, South Africa, Tanzania, Thailand, Uganda, Vietnam, Zambia | 2008-2018 | HPV; PCV; Rotavirus; Measles; Rubella; Polio; HiB; Hepatitis B; BCG; Diphtheria; Pertussis; Tetanus; Meningococcal; Yellow Fever; Cholera; Japanese Encephalitis; Malaria | Low | Community participation; Two-way communication; Mass communication; Community-based activities; Capacity strengthening; Research and data | Efficient response; Community-led actions; Trusted actors and mechanisms; Safe and protective behaviours | Thorough planning and community partnerships are essential for successful introductions and equitable coverage |
| Guillaume et al. (2022) | Haiti | 2000-2021 | HPV | Medium | Mass communication | Efficient response; Trusted actors and mechanisms; Community cohesion | Recommendation of and relationships with health care providers was critical motivator in increasing vaccination uptake. Women listened to recommendations of physicians, CHWs, & ancillary health care staff to guide vaccination decision making. Social networks functioned as both barriers & facilitators to vaccine uptake. Family members & elders were consulted prior to vaccination decision. Influential sources of health information included radio, television, churches, & word of mouth |
| Ismail et al. (2022) | Nigeria, South Sudan, Afghanistan, Cameroon, Haiti, Somalia | 2021 | Routine childhood vaccines | High | Community participation; Two-way communication; Mass communication; Capacity strengthening | Efficient response; Trusted actors and mechanisms; Safe and protective behaviours; Community cohesion; Community-led actions | Sector-specific roles improve accountability in conflict settings. Multi-channel approaches increase preparedness and resilience. Two-way communication; Community participation is vital for adapting interventions quickly. Capacity strengthening improves system resilience. |
| Jain et al. (2022a) | Afghanistan, Bangladesh, Ethiopia, Ghana, Guatemala, Honduras, India, Indonesia, Kenya, Madagascar, Mexico, Nepal, Nigeria, Pakistan, South Africa, Tanzania, Uganda, Zambia, Zimbabwe | 2020 | Diphtheria; Tetanus; Pertussis; Polio; Measles; Routine childhood vaccines | High | Capacity strengthening; Community participation; Two-way communication | Efficient response; Trusted actors and mechanisms; Community-led actions | Effective community engagement involving religious leaders improved trust and vaccine uptake. Programs that involved community buy-in or development of new community-based structures were more effective. Tailored messaging and addressing misinformation were crucial in achieving better immunization coverage​ |
| Jain et al. (2022b) | Afghanistan, Bangladesh, Ethiopia, Ghana, Guatemala, Honduras, India, Indonesia, Kenya, Madagascar, Mexico, Nepal, Nigeria, Pakistan, South Africa, Tanzania, Uganda, Zambia, Zimbabwe | 2020 | Diphtheria; Tetanus; Pertussis; Polio; Measles; Routine childhood vaccines | High | Community-based activities; Two-way communication; Capacity strengthening | Efficient response; Trusted actors and mechanisms; Community cohesion; Community-led actions; Safe and protective behaviours | Tailored communication addresses vaccine hesitancy effectively. Promoting equity ensures marginalized groups are reached. Building trust through two-way communication improves uptake. Capacity strengthening supports sustainable vaccination. |
| Jain et al. (2024) | Multiple LMICs | 2021 | Routine childhood vaccines | Medium | Community participation; Capacity strengthening; Research and data | Efficient response; Safe and protective behaviours; Trusted actors and mechanisms | Addressing misinformation leads to better vaccine outcomes. Caregiver and community participation improves trust. Equity in interventions ensures vulnerable populations are included. Strengthening local systems boosts accountability and effectiveness. |
| Karanja-Chege (2022) | Kenya | 2013-2019 | HPV | Low | Mass communication; Community participation; Capacity strengthening; Research and data; Two-way communication; Community-based activities | Efficient response; Trusted actors and mechanisms; Community cohesion; Community-led actions | Teachers’ knowledge & attitude plays major role in success of school-based vaccination programs, & should be involved in provision of information to increase confidence in vaccine. Increased acceptability of school-based HPV vaccine among parents & caregivers as teachers seen as trusted stakeholders & schools as safe havens. CHV were essential in reaching girls not enrolled in schools, especially in marginalized communities.  Advocacy, social mobilization & communication activities conducted using social & mainstream media with information tailored to varying literacy levels. Information, Education & Communication materials developed in English & Swahili targeting key audiences with simple messages. Knowledge gaps were largest contributor to vaccination refusal. Most influential vaccine opponents were Catholic Church, who cited misinformation to raise doubts about safety & efficacy. Multi-stakeholder advocacy strategies aimed at countering misinformation & disseminating facts about safety & efficacy. Sharing of real-world stories from other countries on impact of HPV vaccines on cervical cancer.  Increased demand among some girls. Uptake of 1st dose improved over time with focused advocacy using CHWs. Poor response in areas with higher social media use, which amplified misinformation. |
| Majekodunmi et al. (2022) | Malawi, Nigeria, Ghana, Kenya, Burkina Faso, Guinea, South Africa, Democratic Republic of the Congo, Burundi, Madagascar, Mozambique, Rwanda, Tanzania, Zambia, Zimbabwe, Ethiopia, Liberia, Sierra Leone | 2017 | Measles | High | Mass communication; Capacity strengthening | Efficient response; Trusted actors and mechanisms | Several different factors inﬂuencing measles vaccine coverage were identiﬁed and were grouped into four main areas: immunization system, information and communication, family characteristics and parental attitudes and knowledge. Examples of communications interventions that help improve coverage are presented in the results and discussion, such as communication via suitable channels (community and religious leaders, female leaders, health workers and mass media campaigns), local health worker capacity strengthening, and adoption of different communication formats (house-to-house visits, SMS reminders, use of community stakeholders and authority figures), and tailoring messaging to local contexts. |
| Naidoo et al. (2023) | Ethiopia, Ghana, Nigeria, Somalia, South Africa, Democratic Republic of the Congo, Mozambique, Uganda, Libya, Cameroon, Zimbabwe, Kenya, Egypt, Botswana, Guinea | 2022 | COVID-19 | High | Community-based activities; Community participation; Capacity strengthening; Mass communication; Two-way communication; Research and data | Efficient response; Trusted actors and mechanisms; Community cohesion | Factors that promoted vaccine acceptance included conﬁdence in the COVID-19 vaccines and the desire to protect people. Barriers to uptake were often related to information, trust and knowlege, including concerns about potential side effects, vaccine ineffectiveness, a perceived lack of information. However, structural barriers and inaccesibility of vaccines also influenced COVID-19 vaccine uptake. |
| O’Rourke et al. (2023) | Afghanistan, Burundi, Cameroon, Democratic Republic of the Congo, El Salvador, Ethiopia, India, Madagascar, Nigeria, Pakistan, Tanzania, Tajikistan, Zambia | 2022 | BCG; Diphtheria; HiB; Hepatitis B; Measles; Mumps; Pertussis; PCV; Polio; Rotavirus; Rubella; Tetanus; Varicella; Yellow Fever | Medium | Mass communication; Two-way communication; Capacity strengthening; Community participation | Efficient response; Trusted actors and mechanisms; Community cohesion | Education for, and collaboration between, caregivers, healthcare workers and community groups significantly increased vaccination uptake. Non-material incentives also improved trust and coverage in vulnerable populations and motivated caregivers to vaccinate children​. |
| Oketch et al. (2023) | Nigeria, South Africa, Kenya, Uganda, Cameroon, Mali, Tanzania | 2022 | HPV | High | Community participation; Research and data; Two-way communication | Efficient response; Trusted actors and mechanisms; Community cohesion; Safe and protective behaviours; Community-led actions | Vaccine communication (face-to-face communication, use of information, education and communication (IEC) materials, media) needs to pass critical information about the vaccine and its benefits while also addressing the concerns of the community about the vaccine. Communication needs to be sustained to encourage the target population to be fully vaccinated, to target the key members of the community including teachers, school management boards, community and religious leaders, and the parents, and to ensure community ownership. |
| Oliver-Williams et al. (2017) | Bangladesh, India, Kenya, Malawi, Nigeria, Pakistan, Philippines, Zambia, Zimbabwe | 2017 | Diphtheria; Tetanus; Pertussis | High | Community participation | Efficient response; Trusted actors and mechanisms; Community cohesion; Community-led actions | Mobile health technologies, such as SMS reminders to families regarding vaccination or educational tools for health workers, are flexible and widely available tools that can be utilized in myriad ways to improve vaccination uptake. Further research is needed to determine the most effective mHealth interventions and to refine their use. Overall, there is preliminary evidence to support the use of mHealth technology to increase vaccination coverage in LMIC. |
| Omoniyi & Williams (2020) | Nigeria, Iraq, Pakistan, Afghanistan, India, Zimbabwe, Guatemala, Bangladesh, China | 1996-2019 | Routine childhood vaccines | High | Two-way communication; Capacity strengthening; Research and data; Community participation | Efficient response; Trusted actors and mechanisms; Community cohesion; Community-led actions | Communication/Education: In predominantly traditional Muslim societies, education & engagement of traditional & religious leaders as advocates for immunisation helped enhance community acceptance of vaccination. In India absence of leadership involvement hindered uptake. Increased awareness impacted demand & supply sides of vaccination. In Nigeria, vaccine completion rates higher in children of mothers that received focused, short duration (5 minutes) education on immunisation, rather than longer (10-15 minutes) general health promotion messages (which included information on vaccination). In Nepal, focused messages believed to aid retention & recall, while longer sessions led to information overload & reduced focus. Educational programmes should critically appraise communities local interventions & culture before proposing what may be perceived as alien practices. Provide space for other strategies to be identified during discussions to identify concerns of caregivers. Targeting caregivers with home visits & radio/television messages enhanced access to some population members who might otherwise be missed. Effective mechanisms include pictorial messages & home visits.   Reminder-Type Interventions: In Zimbabwe, higher vaccination coverage & reduced delays with reminder text messages sent in local language. In high vaccination setting (Guatemala), no impact found of reminders. In rural Pakistan, brief centre-based maternal education during each immunisation visit were successful in increasing follow-up immunisation visits.  Social/Community Mobilisation: Interventions engaged non-health workers to reach mothers within communities resistant to vaccination, & identify/refer eligible children for vaccination. Higher vacc. uptake with home visits for direct personal communication to dispel myths, educate mothers & vaccinate children. Convenience & efficiency of having vaccines administered at home, and involvement of fathers in decision-making, contributed to gains.  Multi-pronged Interventions: Intervention package (extended EPI service schedule; training for service providers; screening tool to identify immunisation needs among clinic attendants, & EPI support group for social mobilisation) produced significant improvements in vaccination coverage in hard-to-reach children, & led to reduced dropout rates. For multi-pronged packages to be used routinely, necessary to identify which combination of interventions produces greatest impact at lowest cost, so that implementation sustainable in long run. |
| Owoyemi et al. (2021) | Democratic Republic of the Congo | 2018-2020 | EVD | Medium | Mass communication; Two-way communication; Community participation; Community-based activities; Research and data | Efficient response; Trusted actors and mechanisms; Community cohesion | Engagement of locals for service delivery necessary for community vaccination strategy. |
| Oyo-Ita et al. (2023) | Afghanistan, China, Côte d'Ivoire, Ethiopia, Georgia, Ghana, Guatemala, Honduras, India, Indonesia, Kenya, Mali, Mexico, Nepal, Nicaragua, Nigeria, Pakistan, Rwanda, Zimbabwe | 2021 | Diphtheria; Tetanus; Pertussis; Polio; BCG; Measles; Mumps; Rubella; Hepatitis B | High | Community participation; Mass communication; Research and data; Capacity strengthening; Two-way communication | Efficient response; Trusted actors and mechanisms; Community-led actions | Combining health education, community mobilization, and digital reminders was effective in increasing vaccine adherence. Involvement of community leaders was effective in improving vaccine uptake. Cash transfer incentives further enhanced uptake​. |
| Parsekar et al. (2024) | Afghanistan, Bangladesh, Brazil, Burundi, Cameroon, China, Colombia, Côte d'Ivoire, Democratic Republic of the Congo, Ethiopia, Georgia, Ghana, Guatemala, Guinea, Haiti, Honduras, India, Indonesia, Kenya, Madagascar, Malawi, Mali, Mexico, Nicaragua, Nigeria, Pakistan, Palestine, Rwanda, Senegal, South Africa, Tanzania, Uganda, Zambia, Zimbabwe | 2020 | Diphtheria; Tetanus; Pertussis; Polio; Routine childhood vaccines | Medium | Community participation; Two-way communication | Community cohesion; Trusted actors and mechanisms; Community-led actions; Efficient response; Safe and protective behaviours | Two-way communication helps address social barriers. Strengthening local capacity builds long-term trust. Community participation empowers local leaders. Localized interventions ensure sustainability and better outcomes. |
| Saeterdal et al. (2014) | India, Pakistan | 2012 | Diphtheria; Tetanus; Pertussis; Measles | High | Community-based activities; Capacity strengthening; Research and data; Two-way communication; Community participation | Efficient response; Trusted actors and mechanisms; Community cohesion | Two community engagement interventions (community information and question sessions in India, and community planning committees in Pakistan) led to improved knowledge of vaccines, immunisation status, and caregiver attitudes towards vaccines, but made no difference on mother involvement in household vaccination decision-making. |
| Singh et al. (2018) | India | 2017 | Routine childhood vaccines | Medium | Community-based activities; Community participation | Efficient response; Trusted actors and mechanisms | Qualitative evidence that the following interventions have increased immunization coverage in India: Education and awareness; Reminder or recall; Outreach healthcare services; Incentives; Multiple interventions (extended service hours, training of service providers, immunization during health facility visits; community volunteer groups). |
| Sinuraya et al. (2024) | Indonesia | 2018-2021 | COVID-19; Measles; Rubella; Polio; Diphtheria; Pertussis; Tetanus; HiB; HPV | Medium | Community participation; Two-way communication; Mass communication | Trusted actors and mechanisms; Community cohesion; Safe and protective behaviours | Drivers include misinformation, trust, cultural and access constraints; context-tailored engagement needed |
| Tilahun et al. (2020) | Ethiopia | 1993-2018 | Routine childhood vaccines | Medium | Community-based activities; Community participation | Efficient response; Trusted actors and mechanisms; Community-led actions; Community cohesion | Positive: Immunization uptake dependent on major factors: caretaker behaviour, family characteristics, & communication. Mothers knowledge significantly associated with immunization coverage. Children whose mothers had good knowledge on immunization & VPDs, and access to media, predictors of full vaccinated. Health Development Army (HDA) network & regular meetings between community & health system actors identified as potential platform to harness community engagement. HDA ensured greater involvement of individuals & communities in moving from supply-driven to demand-driven immunization services.   Negative: Households not regularly (monthly) visited by Health Extension Workers (HEWs), poor participation in women’s developmental groups, & poor knowledge of child immunization. Incorrect perception on contraindications & vaccine side effects. Forgetting appointment date, lack of awareness, fear of side effects, & hearing misinformation about vaccines. Poor counselling of mothers, unsupportive provider-client relationship, & lack of system for tracking defaulters. |
| Tsu et al. (2014) | Bhutan, Bolivia, Brazil, Cambodia, Cameroon, Haiti, India, Lesotho, Nepal, Rwanda, South Africa, Tanzania, Uganda, Vietnam | 2013 | HPV | Medium | Community-based activities; Capacity strengthening; Research and data; Two-way communication; Community participation | Efficient response; Trusted actors and mechanisms | Community outreach and mobilization issues including informed consent, messages and channels, endorsement and support, and timing of mobilization efforts are relevant factors for the uptake of HPV vaccines among adolescents in low resource settings, and may help inform efforts to improve uptake of similar vaccines, such as for HIV. |
| Vouking et al. (2017) | Ethiopia, Kenya, Nigeria, Côte d'Ivoire | 1994-2015 | Tetanus | High | Community-based activities; Community participation | Efficient response; Trusted actors and mechanisms; Community-led actions; Community cohesion | Co-management & co-design integral to success. Lack of community participation was crucial constraining factor. Must enhance communication to address safety & effectiveness concerns. Women’s lower social status negatively affects access to vaccines due to weak decision-making power over resources & lack of autonomy. Coverage could be improved by involving fathers & communities in immunization activities. |
| Alum et al. (2025) | Ghana, Kenya, Malawi | 2014-2024 | Malaria | Medium | Community participation; Two-way communication; Mass communication; Research and data | Trusted actors and mechanisms; Safe and protective behaviours; Efficient response | Positions RTS,S within SSA control efforts, highlighting community engagement and context-specific messaging as central to acceptance and completion. |
| Bello et al. (2011) | Nigeria, India, Uganda, Tanzania, South Africa, Rwanda | Not stated | HPV | Medium | Community participation; Mass communication; Two-way communication | Trusted actors and mechanisms; Community cohesion; Safe and protective behaviours; Community-led actions | Engaging schools, faith leaders and providers alongside clear public information is pivotal for HPV uptake in resource-limited settings. |
| Ewongwo et al. (2024) | Brazil, China, Guinea, India, Kenya, Malaysia, Niger, Nigeria, Peru, Rwanda, Tanzania, Thailand, Uganda, Vietnam | 2006-2021 | HPV | Medium | Research and data; Community participation; Two-way communication | Trusted actors and mechanisms; Safe and protective behaviours; Efficient response | Synthesises pathways to broaden HPV access, stressing provider counselling and community dialogue to support initiation and completion. |
| Eze et al. (2025) | Nigeria | 1995-2020 | Polio | High | Community participation; Community-based activities; Mass communication; Capacity strengthening | Efficient response; Community-led actions; Trusted actors and mechanisms; Safe and protective behaviours | Community mobilisation, house-to-house strategies and service strengthening linked to reductions in polio transmission and improved coverage. |
| George et al. (2025) | Angola, Bangladesh, Benin, Botswana, Burkina Faso, Burundi, Cabo Verde, Cameroon, Central African Republic, Chad, Comoros, Congo, Democratic Republic of the Congo, Djibouti, Eritrea, Eswatini, Ethiopia, Gabon, Gambia, Ghana, Guinea, Guinea-Bissau, Kenya, Liberia, Libya, Madagascar, Malawi, Mali, Mauritania, Mozambique, Namibia, Niger, Nigeria, Rwanda, Senegal, Seychelles, Sierra Leone, Somalia, South Sudan, Sudan, Tanzania, Togo, Uganda, Zambia, Zimbabwe | 2000-2024 | Routine childhood vaccines; COVID-19 | High | Community-based activities; Community participation; Two-way communication; Research and data | Trusted actors and mechanisms; Community cohesion; Efficient response; Community-led actions | Refugee-focused programmes perform best when co-designed with communities and supported by trusted intermediaries and context mapping. |
| Kane et al. (2012) | Bhutan, China, India, Indonesia, Malaysia, Morocco, Peru, Rwanda, Tanzania, Uganda, Vietnam | 2007-2012 | HPV | Low | Community participation; Mass communication; Capacity strengthening | Trusted actors and mechanisms; Community cohesion; Safe and protective behaviours | Early implementation lessons highlight the role of schools, local leaders and trained providers in HPV programme acceptance. |
| Lambo & Nagulesapillai (2012) | Pakistan | 2000-2011 | Tetanus | Low | Community-based activities; Mass communication; Community participation | Efficient response; Trusted actors and mechanisms; Community-led actions | Progress toward NNT elimination included outreach to pregnant women and engagement of community structures. |
| Obregon et al. (2009) | India | 1995-2009 | Polio | Medium | Mass communication; Two-way communication; Community participation | Trusted actors and mechanisms; Efficient response; Community cohesion; Safe and protective behaviours | Health communication for polio eradication underscored community dialogue and locally tailored messages to counter rumours and refusals. |
| Reda et al. (2025) | Bangladesh | 2020-2023 | COVID-19 | Medium | Community-based activities; Community participation; Two-way communication | Safe and protective behaviours; Trusted actors and mechanisms; Community cohesion | Trust, tailored risk communication and involvement of community representatives were key to protective behaviours and vaccination among FDMNs. |
| Vadrevu et al. (2024) | India | 2010-2020 | Routine childhood vaccines; COVID-19 | High | Research and data; Community participation; Two-way communication | Efficient response; Trusted actors and mechanisms; Community-led actions | Maps linkages between BeSD domains and uptake, highlighting community co-design and feedback loops as levers for programme improvement. |
