# Supplementary material for: Community engagement for vaccine delivery in low- and middle-income countries and humanitarian settings: A scoping umbrella review
Source: PLOS Glob Public Health. 2026 Apr 24;6(4):e0006307. doi: 10.1371/journal.pgph.0006307 (PMC13108762; doi:10.1371/journal.pgph.0006307)
Supplement: S1 Fig — (DOCX) [file pgph.0006307.s006.docx]

## S1 Fig: Community Engagement Impact Framework (*Source: International Federation of Red Cross and Red Crescent Societies (IFRC))*


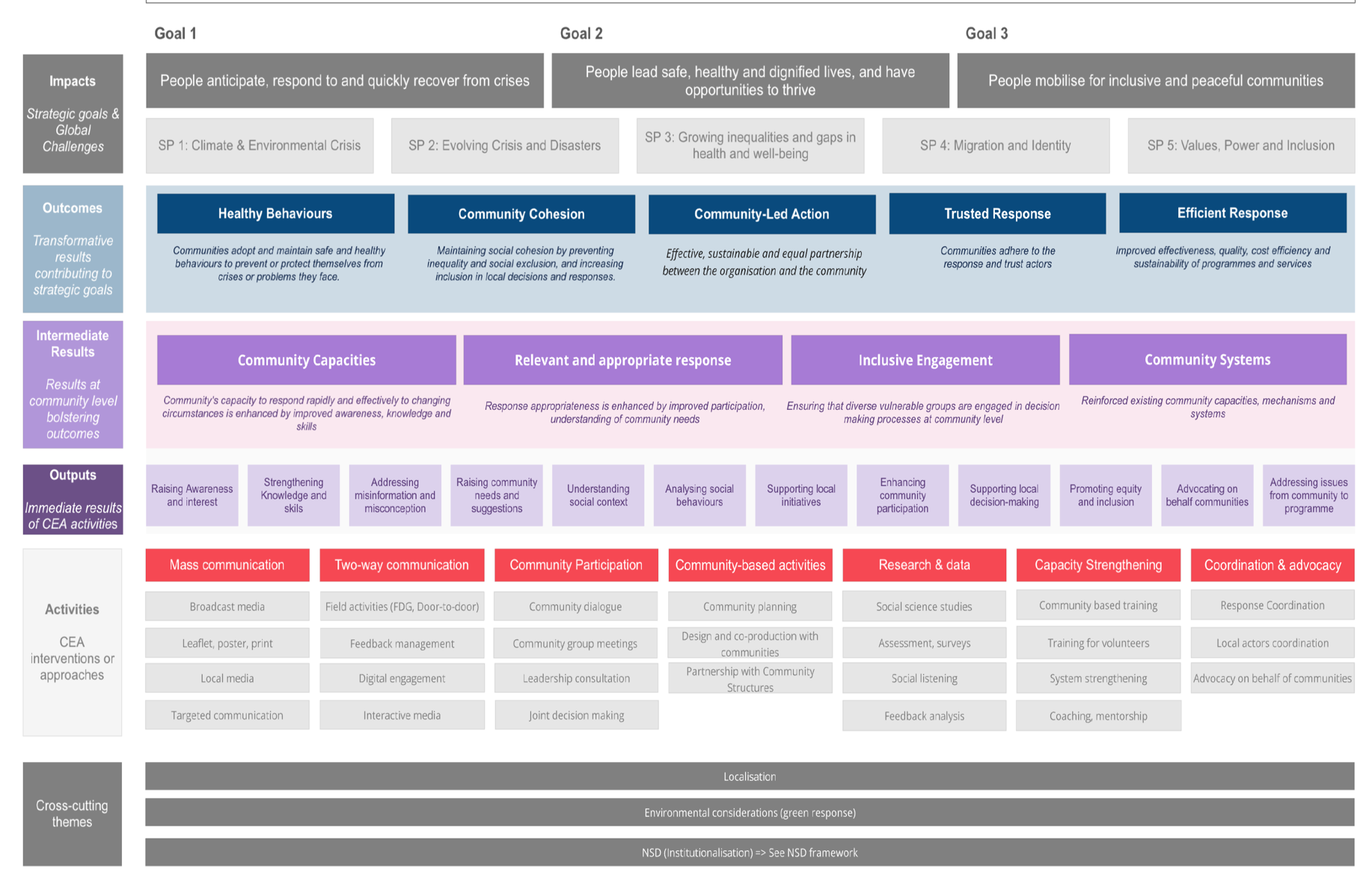


Source: IFRC
